# Uncovering the hidden players in Lepidoptera biology: the heritable microbial endosymbionts

**DOI:** 10.7717/peerj.4629

**Published:** 2018-05-08

**Authors:** Anne Duplouy, Emily A. Hornett

**Affiliations:** 1 Organismal and Evolutionary Biology Research Program, University of Helsinki, Helsinki, Finland; 2 Department of Zoology, University of Cambridge, Cambridge, UK

**Keywords:** Symbiosis, *Wolbachia*, Conservation, Climate change, Butterfly, Moth, Microbes

## Abstract

The Lepidoptera is one of the most widespread and recognisable insect orders. Due to their remarkable diversity, economic and ecological importance, moths and butterflies have been studied extensively over the last 200 years. More recently, the relationship between Lepidoptera and their heritable microbial endosymbionts has received increasing attention. Heritable endosymbionts reside within the host’s body and are often, but not exclusively, inherited through the female line. Advancements in molecular genetics have revealed that host-associated microbes are both extremely prevalent among arthropods and highly diverse. Furthermore, heritable endosymbionts have been repeatedly demonstrated to play an integral role in many aspects of host biology, particularly host reproduction. Here, we review the major findings of research of heritable microbial endosymbionts of butterflies and moths. We promote the Lepidoptera as important models in the study of reproductive manipulations employed by heritable endosymbionts, with the mechanisms underlying male-killing and feminisation currently being elucidated in moths and butterflies. We also reveal that the vast majority of research undertaken of Lepidopteran endosymbionts concerns *Wolbachia*. While this highly prevalent bacterium is undoubtedly important, studies should move towards investigating the presence of other, and interacting endosymbionts, and we discuss the merits of examining the microbiome of Lepidoptera to this end. We finally consider the importance of understanding the influence of endosymbionts under global environmental change and when planning conservation management of endangered Lepidoptera species.

## Introduction

Symbiosis was originally described as the living together of dissimilar organisms in an intimate association (de Bary, 1879). This broad term is commonly used to encompass relationships between two or more organisms that range from parasitic, through commensal (one party gains a benefit, whilst the other is not significantly affected) to mutualistic (both parties benefit). We now know that the nature of an association is often much more complex, and varies greatly depending on factors such as the local environment, the host genetic background or condition, and the longevity of the relationship. Thus it is perhaps now more pertinent to understand symbiosis as an interaction in which two or more organisms of different species are in a persistent relationship, with no pre-conceived idea of the nature of the interaction.

One of the most intimate associations between species is that between a host organism and a microbial endosymbiont (a symbiont living within the body of its host). This lifestyle substantially affects the relationship between the two parties as survival and reproduction of host and microbe are intrinsically linked. Where the endosymbiont is intracellular—residing within the cytoplasm of host cells—it is predominantly inherited through the female line (although intrasperm paternal transmission has also been described (Watanabe et al., 2014)). Such maternal inheritance produces selection upon the symbiont to favour the cytoplasmic lineage of the host (in essence the females)—a phenomenon that has resulted in the evolution of remarkable manipulations of host reproductive biology, including sex-ratio distortion (O’Neill, Hoffmann & Werren, 1998; Bandi et al., 2001; Engelstädter & Hurst, 2009). With increasing pace, evidence is gathering that diverse endosymbionts interact with many aspects of arthropod host biology including host reproduction (Werren, Zhang & Guo, 2004), development (Fraune & Bosch, 2010), immunity (Gross et al., 2009; Nyholm & Graf, 2012), behaviour (Dion et al., 2011), body colour (Tsuchida et al., 2010), nutritional stress resistance (Brownlie et al., 2009), pathogen load (Graham & Wilson, 2012), dispersal (Goodacre et al., 2009), host plant specialisation (Leonardo & Muiru, 2003), thermal tolerance (Dunbar et al., 2007), nutrition (Douglas, 1998) and metabolism (McCutcheon, McDonald & Moran, 2009). Furthermore, symbiosis has been purported to be a key factor underlying natural variation, as well as an instigator of novelty and a promoter of speciation (Margulis & Fester, 1991; Brucker & Bordenstein, 2012).

Since the advent of the diagnostic PCR assay in the mid-1980s, organisms can be routinely screened for known endosymbionts. As a consequence of this development and recent advancements in genomics and bioinformatics (including high-throughput amplicon sequencing of microbial genes and metagenomics), we now recognise that all organisms are infected by a diverse range of microbes, including viruses, fungi and bacteria, and that many arthropods carry heritable endosymbionts. A recent study estimated that 52% of terrestrial arthropod species are infected with the intracellular bacteria *Wolbachia*, with a further 24% and 13% species infected with *Cardinium* and *Rickettsia* bacteria, respectively (Weinert et al., 2015). How species initially acquire heritable endosymbionts is not yet fully understood. While phylogenetic evidence suggests that horizontal transfer of endosymbionts on an evolutionary scale must be common, many barriers—ecological, geographical and physiological—exist that perturb the spread of endosymbionts between species and prevent the formation of novel symbioses. Successful transfer of an endosymbiont between species depends on the ability of the microbe to first enter and then survive in a novel host environment, followed by successful migration to the host germline to ensure propagation. The symbiont must then be able to invade the host population, or at least be maintained at low frequency. Thus the ‘fit’ between a host and symbiont can be quite specific, with host biology playing an important role in the ability of the symbiont to thrive in the novel species. Failure in the formation of persistent associations may also be due to the endosymbiont causing harm to their new hosts (Hutchence et al., 2011). Where movement of heritable endosymbionts has been observed, it is often via ecological connectors such as shared host food sources (Huigens et al., 2000; Duron, Wilkes & Hurst, 2010; Caspi-Fluger et al., 2012; Chrostek et al., 2017) or common symbiont-vector parasites or parasitoids (Heath et al., 1999; Vavre et al., 1999; Huigens et al., 2004; Jaenike et al., 2007; Gehrer & Vorburger, 2012). Horizontal transfer is perhaps more successful between related hosts (Russell et al., 2009); it has been suggested that within *Acraea* butterflies, *Wolbachia* has moved between species either via a common parasitoid, or through hybridisation and subsequent introgression. It is also possible that the different species inherited the bacteria from a recent common ancestor (Jiggins et al., 2000b).

The Lepidoptera are remarkably diverse and widely recognisable, encompassing butterflies and moths that are economically and ecologically important. While many aspects of Lepidopteran biology have been well studied, it is only recently that the pervasiveness of host-associated microbes in this group has been appreciated. Heritable endosymbionts have been the subject of several reviews (Bandi et al., 2001; Moran, McCutcheon & Nakabachi, 2008; Duron & Hurst, 2013), and here we focus upon studies of these influential elements in the Lepidoptera. Butterflies and moths are particularly important in the study of heritable endosymbionts due to the Lepidoptera sex determination system. In contrast to most other arthropod groups, the female is the heterogametic sex (females have one Z and one W sex chromosome, males have two Z chromosomes). The mechanisms and repercussions of reproductive manipulations caused by inherited microbial endosymbionts, which are commonly observed in butterflies and moths, are therefore likely to be very different from that observed in arthropods with alternative sex determination systems. Furthermore, in the Lepidoptera, heritable endosymbiont prevalence is commonly very high, and vertical transmission of the infection is often near perfect. Together with the maternal inheritance of intracellular endosymbionts such as *Wolbachia*, this creates linkage of the infection not only with similarly maternally inherited host mitochondria, but also with the female W chromosome. Formation of this wider co-inherited network may have implications for host genetic diversity and even the sex determination system itself.

In this review, we summarise the main body of research that has been conducted to date in order to form a springboard for future work and to emphasise to researchers from traditionally disparate fields as ecology, genomics and conservation, that in order to fully understand the biology of an organism, one must take into account its endosymbionts. For clarity this review is divided into areas of current research: (1) Manipulation of host reproduction; (2) impact upon host fitness; (3) symbiont-mediated protection; (4) host genetics and (5) behavioural modification. We then highlight outstanding questions and future directions, including consideration of the influence of endosymbionts under global environmental change, and in species of conservation concern.

### Survey methodology

The authors have drawn upon knowledge gained from over a decade in butterfly-endosymbiont research. Extensive literature searches were performed using repositories such as NCBI PubMed and Google Scholar, and using keywords including ‘endosymbiont,’ ‘microbe’ and ‘heritable symbiont,’ along with ‘butterfly,’ ‘moth’ and ‘Lepidoptera.’ Social media platforms such as Twitter provided a useful tool to obtain up to date information of relevant publications. Research on heritable endosymbionts of arthropods in general was also gathered with the aim to provide information about areas in heritable endosymbiont-arthropod research that is lacking for Lepidoptera. Particular effort was made to compile a comprehensive list of butterfly and moth species that are published as infected with heritable endosymbionts.

### The influence of heritable microbial endosymbionts on Lepidopteran biology

Concordant with general insect surveys, the Lepidoptera are commonly infected with heritable microbial endosymbionts. In an early screen of Panamanian arthropods, *Wolbachia* was detected in 16.3% of the 43 Lepidoptera species tested (Werren, Windsor & Guo, 1995). Further surveys identified *Wolbachia* in 29% of 24 species of *Acraea* butterflies from Uganda (Jiggins et al., 2001), 45% of 49 species of butterflies studied in Japan (Tagami & Miura, 2004), 50% of 56 Indian butterfly species (Salunke et al., 2012), 58.3% of 120 Lepidoptera species in West Siberia (Ilinsky & Kosterin, 2017) and 79% of 24 species of African *Bicyclus* butterflies (Duplouy & Brattström, 2017). Additionally, in a broad survey of ants, moths and butterflies (specifically Lycaenidae and Nymphalidae) for five heritable symbionts, *Wolbachia* (39 of 158 species) and *Spiroplasma* (five of 200 species) were found to infect Lepidopteran species (Russell et al., 2012). In general, these estimates are likely to be highly conservative, due to the presence of undetected low frequency infections, geographical and temporal variation in infection, tissue-specificity and PCR false negatives. Geographic structure in infection incidence and prevalence is a particularly important consideration and especially evident in endosymbiont-Lepidoptera systems, e.g. *Wolbachia*–*Hypolimnas bolina* butterflies (Charlat et al., 2005). In a recent survey of published records of *Wolbachia* infections in the Lepidoptera, generalised geographic structure in infection frequency was observed, with lower frequencies towards higher latitudes (Ahmed et al., 2015).

In Tables S1 and S2 we compile a comprehensive list of butterfly and moth species, respectively, reported as carrying heritable endosymbionts from published sources. We find that research of heritable endosymbionts in Lepidoptera is heavily dominated by studies of *Wolbachia* as opposed to that of other infections (*Wolbachia* in 248/253 butterfly species and in 109/115 moth species). While arthropod-infecting endosymbiont diversity is notable, including such divergent taxa as *Rickettsia*, *Spiroplasma*, *Arsenophonus*, *Flavobacteria*, *Cardinium* and the microsporidia, much of the early arthropod endosymbiont literature focused upon the Alphaproteobacteria genus *Wolbachia* (Hertig & Wolbach, 1924). Due to its presence in many agricultural pests and disease vectors, and also owing to the range of reproductive manipulations it employs in the host, *Wolbachia* is still widely, but justly, studied.

Tables S1 and S2 reveal that *Wolbachia* is common across Lepidopteran families, being found in all five families of ‘true’ butterflies (the Papilionoidea), and also in the skippers (Hesperiidae). *Wolbachia* strains have been divided into separate genetic lineages termed supergroups. It is clear from the compiled data that the *Wolbachia* strains carried by Lepidoptera are almost exclusively from supergroups A and B, with B group *Wolbachia* predominating over A group *Wolbachia*. Of species where the *Wolbachia* supergroup has been determined (80/109 moths and 208/248 butterflies), 85% of moth species carry B group and 25% A group; while 79% of butterfly species carry B group *Wolbachia* and 26% carry A group. Note that these data include multiple infections (i.e. some species harbour both A and B strains of *Wolbachia*). These findings concur with research analysing 90 *Wolbachia* strains associated with Lepidoptera: 84% of the strains belonged to supergroup B (76/90), with the remainder (14/90) belonging to supergroup A (Ahmed, Breinholt & Kawahara, 2016). A further study identified 22 *Wolbachia*-infected Lepidoptera species in Japan, 19 of which had infections from supergroup B (86%), with the remaining three from supergroup A (Tagami & Miura, 2004). It is unclear why B group *Wolbachia* are particularly prevalent in the Lepidoptera; is there a greater ‘fit’ between Lepidoptera and B group *Wolbachia*, i.e. are B group *Wolbachia* more likely to become established, or are B group *Wolbachia* those ancestrally associated with the Lepidoptera thus seeding this group stochastically? It is also interesting to note that there appears to be one particularly common strain of *Wolbachia* in Lepidoptera. In a study of 53 Lepidoptera species, 11 species across three families are infected with *Wolbachia* ST41, the next most common strain types (ST40 and ST125) were found in three species each (Ahmed, Breinholt & Kawahara, 2016). Whether *Wolbachia* ST41 is especially adept at moving between species, and/or whether it is particularly successful at establishing and maintaining itself with the host remains to be fully investigated.

The second most common heritable endosymbiont recorded in butterflies and moths is *Spiroplasma*—a bacterial genus belonging to the class Mollicutes (Table S1: 5/253 butterfly species and Table S2: 5/115 moth species). Until such endosymbionts receive the same level of attention as *Wolbachia*, or there is a move towards a generalised metagenomic approach to identify symbiotic microbes, little can be said of the extent of their presence or action in Lepidoptera. However, while there is a propensity for Lepidoptera to be specifically screened for *Wolbachia* infections (thereby creating a bias towards detection of *Wolbachia*), discovery of sex-ratio distorter identity is commonly a phenotype forward investigation, i.e. a sex-ratio bias in progeny or in a population is observed, and then the causative factor is identified. Thus, there should be no bias in the responsible infection found in these studies. Despite this, it appears that Lepidoptera are different from many other groups, e.g. ladybirds, in that *Wolbachia* is almost always responsible for the observed sex-ratio bias. In comparison with other arthropod groups such as the Diptera, Hymenoptera and Hemiptera, heritable endosymbiont diversity does appear to be particularly low in the Lepidoptera. A systematic review (Russell et al., 2012) compiling data of infection screens of arthropods for the heritable endosymbionts *Arsenophonus*, *Cardinium*, *Hamiltonella*, *Spiroplasma* and *Wolbachia*, found that only the latter two genera of bacteria were present in Lepidoptera species (*Spiroplasma*: 5/205, *Wolbachia*: 140/481 species infected). Thus we can say that for *Arsenophonus*, *Cardinium* and *Hamiltonella*, where 263, 183 and 251 Lepidopteran species were assayed respectively, such infections, should they exist at all in Lepidoptera, are remarkably rare. A later compilation of data of arthropods screened for heritable endosymbionts found that *Rickettsia* bacteria were also not commonly found in Lepidoptera, with only one species (an unidentified Noctuidae moth) infected out of 14–32 species (variation in number reported here due to several individuals tested having no taxonomic assignment in the study (Weinert et al., 2015)).

### Manipulation of host reproduction

**Figure 1:**
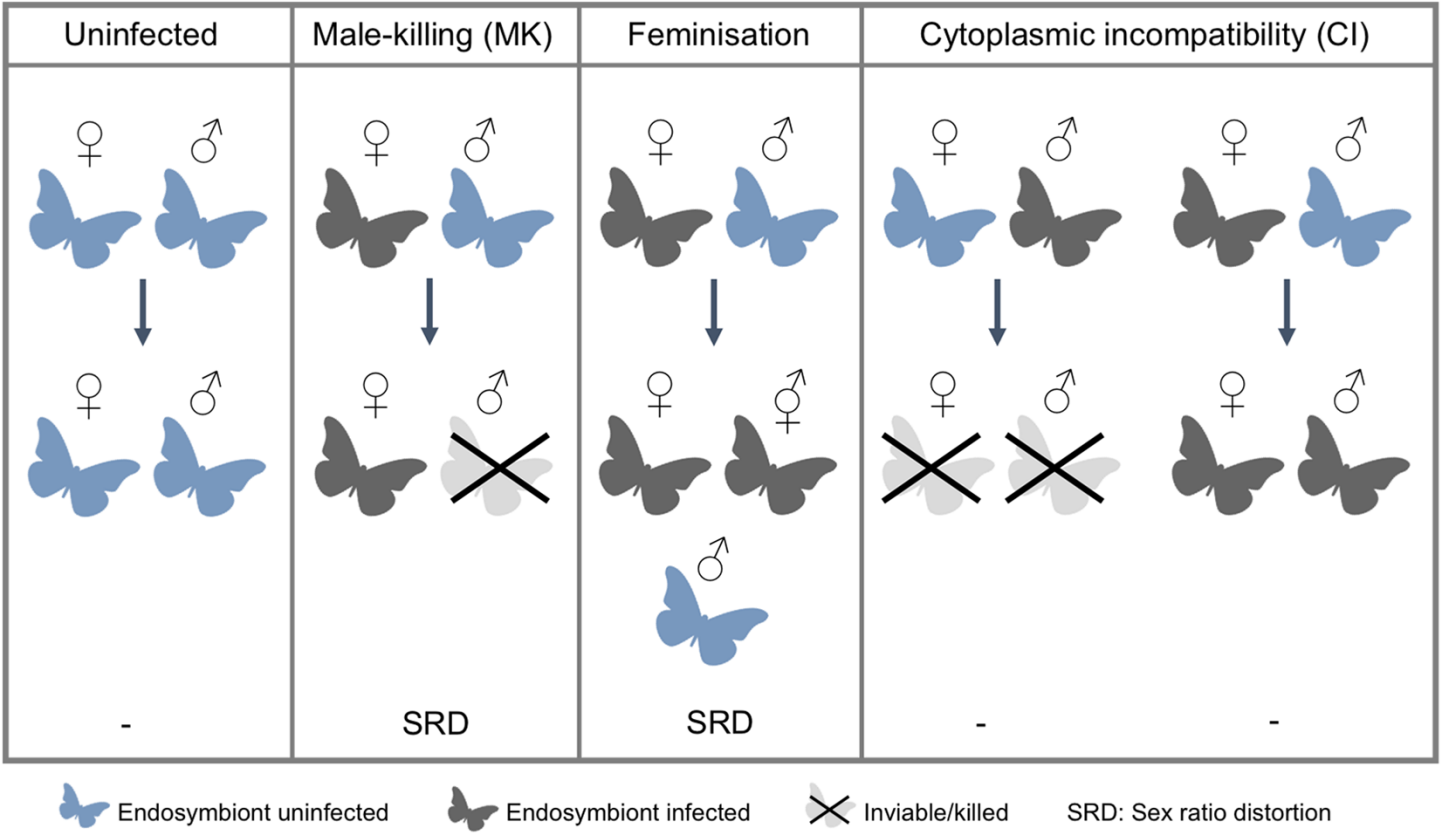
Endosymbiont-induced manipulation of Lepidoptera reproduction. In the Lepidoptera, endosymbionts are currently known to manipulate host reproduction in three ways in order to increase their transmission to the next generation. Male-killing: female hosts infected with male-killing endosymbionts only give rise to infected female offspring, with male offspring dying early in development. Feminisation: female hosts infected with feminising endosymbionts only give rise to infected female offspring, with male offspring having been feminised so that they are genetically male (ZZ) but phenotypically female. Uninfected males may arise through inefficient transmission of the infection. Cytoplasmic incompatibility (CI): crosses between uninfected females and infected males result in few or no viable offspring, as the result of an incompatibility induced by the endosymbiont in the male. Infected females are able to rescue this incompatibility and hence are able to produce viable (infected) offspring when mated with infected males. For male-killing and feminisation the endosymbiont acts as a sex-ratio distorter, creating a female-bias in the offspring, and potentially in the population if the infection is highly prevalent.

The Lepidoptera are becoming model systems for the study of endosymbiont manipulation of host reproduction. Many species are infected with maternally inherited bacteria that have evolved the ability to alter host reproduction to either increase the proportion of infected females in the population, or increase the reproductive fitness of infected females relative to their uninfected counterparts. In Lepidoptera endosymbionts are currently known to manipulate host reproduction in three ways: through male-killing (MK), feminisation and cytoplasmic incompatibility (CI) (Fig. 1). While these methods facilitate the maintenance of the symbiont in the host population, there are often severe repercussions for host biology and evolution. We provide a list of butterflies and moths that have been recorded as being infected with endosymbionts that manipulate the reproductive biology of the host (Table 1).

**Table 1:** Lepidoptera species carrying heritable endosymbionts that manipulate host reproduction.

	Host	Endosymbiont	Phenotype	Source
**Butterflies**	**Lycaenidae**			
	*Talicada nyseus*	*Wolbachia*	Sex-ratio distortion	Ankola, Brueckner & Puttaraju (2011)
	*Zizina emelina*	*Wolbachia*	MK	Sakamoto et al. (2011)
	**Nymphalidae**			
	*Acraea acerata*	*Wolbachia*	CI	Jiggins et al. (2001)
	*Acraea encedana*	*Wolbachia*	MK	Jiggins et al. (2000a)
	*Acraea encedon*	*Wolbachia*	MK	Jiggins, Hurst & Majerus (1998); Jiggins et al. (2000a)
	*Acraea eponina*	*Wolbachia*	MK	Jiggins et al. (2001)
	*Acraea stoikensis*	*Wolbachia*	MK	Hassan & Idris (2013)
	*Danaus chrysippus*	*Spiroplasma ixodetis*	MK	Jiggins et al. (2000b)
	*Hypolimnas bolina*	*Wolbachia*	MK &/or CI	Dyson, Kamath & Hurst (2002); Charlat et al. (2006); Hornett et al. (2008)
	**Pieridae**			
	*Colias erate poliographus*	*Wolbachia*	CI	Narita, Shimajiri & Nomura (2009)
	*Eurema hecabe*	*Wolbachia*	Feminisation, CI	Narita et al. (2011)
	*Eurema mandarina*	*Wolbachia*	Feminisation, CI	Hiroki et al. (2002, 2004)
**Moths**	**Crambidae**			
	*Ostrinia furnacalis*	*Wolbachia*	MK	Kageyama et al. (2002)
	*Ostrinia orientalis*	*Wolbachia*	Sex-ratio distortion	Kageyama et al. (2004)
	*Ostrinia scapulalis*	*Wolbachia*	MK	Kageyama & Traut (2004)
	*Ostrinia zaguliaevi*	*Wolbachia, Spiroplasma ixodetis*	MK	Kageyama et al. (2004); Tabata et al. (2011)
	*Ostrinia zealis*	Undefined agent	Sex-ratio distortion	Kageyama et al. (2004)
	**Erebidae**			
	*Lymantria dispar*	Undefined agent	MK	Higashiura, Ishihara & Schaefer (1999)
	**Noctuidae**			
	*Cerapteryx graminis*	*Spiroplasma sp.*	Sex-ratio distortion	Graham, Hartley & Wilson (2011)
	*Spodoptera exempta*	*Wolbachia*	MK	Graham & Wilson (2012)
	*Spodoptera littoralis*	Undefined agent	MK	Brimacombe (1980)
	**Plutellidae**			
	*Plutella xylostella*	*Wolbachia*	Sex-ratio distortion	Delgado & Cook (2009)
	**Pyrallidae**			
	*Cadra cautella*	*Wolbachia*	CI	Sasaki & Ishikawa (1999)
	*Ephestia kuehniella*	*Wolbachia*	CI	Sasaki & Ishikawa (1999)
	**Tortricidae**			
	*Epiphyas postvittana*	Undefined agent	MK	Geier, Briese & Lewis (1978)
	*Homona magnanima*	RNA virus	Late MK	Morimoto et al. (2001); Nakanishi et al. (2008)

**Notes:**

A list of butterfly and moth species that have been recorded as carrying heritable endosymbionts that manipulate the reproduction of the host. Endosymbiont induced phenotypes are given as MK: Male-killing, Late MK: Male-killing occurring late in development; CI: Cytoplasmic Incompatibility; Feminisation; or Sex-ratio distortion (where further investigation is needed to determine the nature of the sex-ratio bias).

#### Male-killing

Male-killing is particularly well-known in the Lepidoptera. Here, male offspring are killed early in development (most usually as an egg, but also as first instar larvae) producing a female-biased sex ratio within an infected female’s offspring (Fig. 1). Should the male killer infect many females, the host population as a whole may become female-biased. Several hypotheses have been proposed to explain why maternally inherited endosymbionts kill male hosts. If infected females gain a fitness benefit from the death of their male siblings over uninfected females (whose male siblings survive), the infection will invade and spread through the host population. Such benefits may include a reduction in the likelihood of detrimental inbreeding (as there are no brothers with which to mate) or a reduction in competition for resources (as there are half as many siblings with which to compete) (Hurst & Majerus, 1992; Hurst, Hurst & Majerus, 1997). In ladybirds, *Wolbachia*-infected female neonates gain an important first meal by consuming their dead brothers, while uninfected females lack this ready source of nutrients (Elnagdy, Majerus & Handley, 2011). However, in Lepidopteran systems, the relative fitness benefit for infected females remains elusive as many of the species studied lay their eggs singly, thus making the likelihood of inbreeding, sibling egg cannibalism or competition unlikely (e.g. *Danaus chrysippus* (Jiggins et al., 2000a)).

Despite the lack of evidence of any fitness benefit being provided to infected females, MK has been recorded numerous times in the Lepidoptera, possibly due to the readily observable phenotype of all-female broods and the long history of Lepidoptera being collected and reared in captivity. Early work recorded the presence of female-biases in wild-caught collections and captive bred broods in both *Acraea encedon* (Poulton, 1914; Owen, 1965, 1970), and *Hypolimnas bolina* (Poulton, 1923, 1926) butterflies. Later, MK *Wolbachia* was identified as the causative agent in both *A. encedon* (Jiggins, Hurst & Majerus, 1998; Hurst et al., 1999) and *H. bolina* (Dyson, Kamath & Hurst, 2002). We now know that populations of *Acraea* butterflies carry highly prevalent MK *Wolbachia* infections, with more than 80% and 95% of Ugandan *A. encedon* and *A. encedana* females being infected, respectively (Jiggins et al., 2000a; Jiggins, Hurst & Majerus, 2000). The *H. bolina* system has become remarkable due to the extensive spatial and temporal variation in the dynamics of the interaction across the South-east Asian to Eastern Pacific range of the butterfly (Charlat et al., 2005; Hornett et al., 2009). The island of Samoa is particularly notable due to its well-documented history of a highly biased sex ratio of 100 females to every male, caused by 99% of female butterflies being infected with a MK *Wolbachia* (Dyson & Hurst, 2004). It appears that MKs are often found at a particularly high frequency within butterfly populations, contrasting patterns seen in other taxa studied such as the ladybirds, where generally less than 49% of females carry an infection (Hurst & Jiggins, 2000). In the lycaenid *Zizina emelina*, at least one of the two *Wolbachia* strains described in Japanese populations is a MK that rapidly increased in prevalence from 65% to 86% within a three year period (Sakamoto et al., 2011).

The consequences of a highly distorted sex ratio are likely to be large (for discussions of evolutionary consequences see (Charlat, Hurst & Mercot, 2003; Engelstädter & Hurst, 2007)). As perhaps can be expected, one direct effect is that a large number of females remain unmated. In Makerere, Uganda, 94% of *Wolbachia*-infected *A. encedon* females were virgins (Jiggins, Hurst & Majerus, 2000). Of Samoan *H. bolina* 50% of infected females were unmated, with the females that did mate showing significant fertility deficiencies, implying sperm limitation (Dyson & Hurst, 2004). However, despite the detrimental impacts of MKs upon the reproductive biology of their hosts, natural host populations infected with high prevalence infections can persist: the 100:1 female to male sex ratio of the Samoan *H. bolina* population persisted for over 100 years (Dyson & Hurst, 2004). Only recently did the dynamics of this interaction change, with the host evolving resistance of the MK activity (Hornett et al., 2006; Charlat et al., 2007b).

*Wolbachia* are not the only endosymbionts that selectively kill male Lepidoptera. In the nymphalid butterfly, *D. chrysippus*, a *Spiroplasma* bacteria, related to a MK strain previously found in ladybirds, underlies the observed MK (Jiggins et al., 2000a). Similarly, while *Ostrinia* corn borer moths are especially well-known to harbour MK *Wolbachia* strains (i.e. the adzuki bean borer *Ostrinia scapulalis* (Kageyama & Traut, 2004), and the Asian corn borer *O. furnacalis* (Sakamoto et al., 2007)), a MK *Spiroplasma* related to that found in *D. chrysippus* infects the butterbur borer *O. zaguliaevi* (Tabata et al., 2011). Mirroring the pattern seen in *H. bolina*, spatial variation of the MK *Spiroplasma* infection was observed in *D. chrysippus* (Smith et al., 1998; Herren et al., 2007), with 40% of females infected in Uganda vs. 4% in East Kenya (Jiggins et al., 2000a). Intriguingly, in this system infection appears to be correlated with a colour pattern allele. Although the forces generating this correlation are unknown, it may be the case that particular host genotypes are more susceptible to, or more efficient at transmitting, the infection than others (Herren et al., 2007).

**Figure 2:**
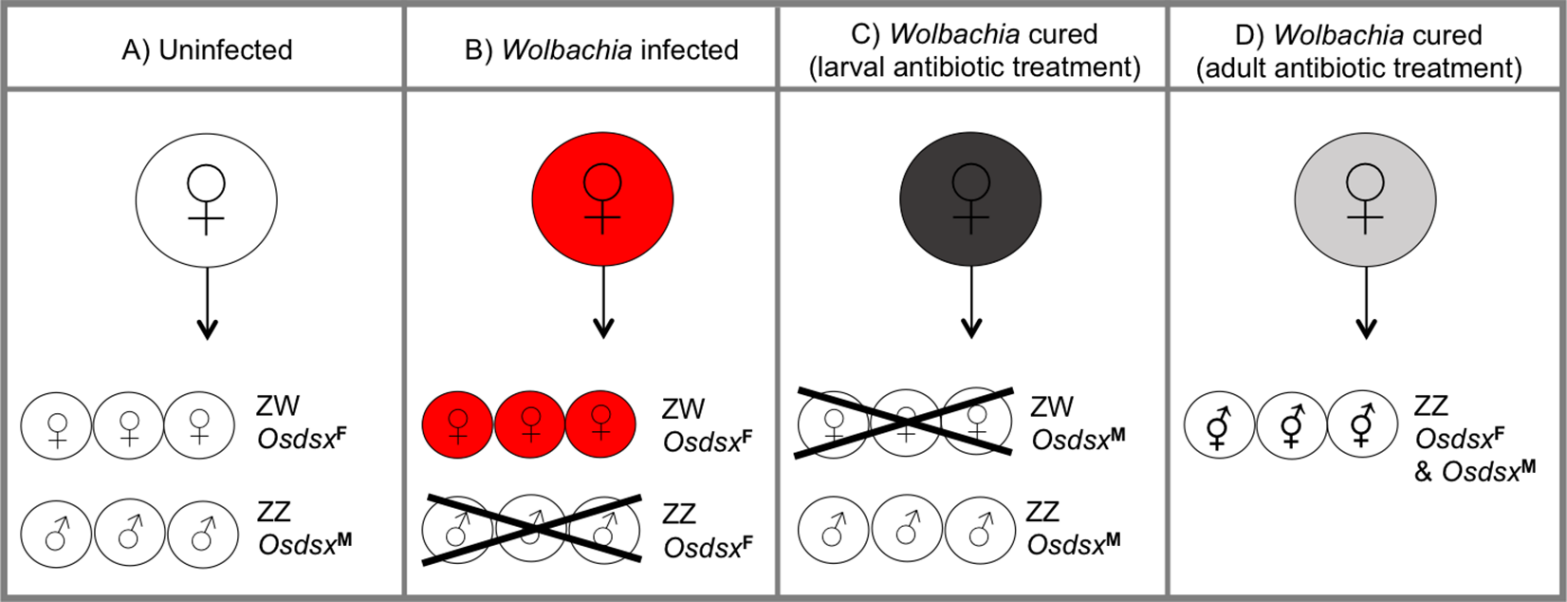
*Wolbachia*-induced male-killing and interference of sex determination in *Ostrinia scapulalis* moths. (A) Uninfected females gives rise to a normal 1:1 sex ratio in progeny: female offspring have ZW sex chromosomes and express the female isoform of the *Ostrinia* homologue of a gene in the sex determination cascade, *doublesex (dsx),* called *Osdsx*^F^; male offspring have two Z sex chromosomes and express the male *dsx* isoform *Osdsx*^M^. (B) *Wolbachia* infected females only give rise to infected female progeny. Male offspring die early in development due to a mismatch between the genotypic sex (ZZ) and phenotypic sex (*Osdsx*^F^). (C) *Wolbachia*-infected females cured of the infection as larvae by antibiotic treatment only give rise to uninfected males. Female offspring die early in development due to a mismatch between their genotypic sex (ZW) and phenotypic sex (*Osdsx*^M^). (D) *Wolbachia*-infected females cured of the infection as adults prior to oviposition by antibiotic treatment give rise to sexual mosaics which have the male ZZ genotype but both *Osdsx*^F^ and *Osdsx*^M^. Note: there are two female isoforms of *dsx in Ostrinia scapulalis*: *Osdsx*^FL^ and *Osdsx*^FS^; these are simplified to *Osdsx*^F^ in this schematic. White circles: uninfected individual; Red circles: *Wolbachia*-infected individual; Dark grey circle, *Wolbachia*-infected female cured as larva; Light grey circle, *Wolbachia*-infected female cured as adult.

In most study systems the precise mechanisms of MK are unclear, and variation across taxa is expected given that MK occurs in arthropods with widely disparate sex determination systems. Dependent on host context several mechanisms have been proposed including defective male chromatin remodelling (*Wolbachia*-infected *Drosophila* (Riparbelli et al., 2012)); targeting the dosage compensation complex (*Spiroplasma*-infected *Drosophila* (Veneti et al., 2005)); damaging the host’s X chromosome to induce embryonic apoptosis (*Spiroplasma*-infected *Drosophila* (Harumoto et al., 2016)), and affecting maternally inherited centrosomes (*Arsenophonus*-infected *Nasonia* wasps: (Ferree et al., 2008)). In a *Wolbachia*-infected moth, *O. scapulalis*, MK is unusual in that males (genotype ZZ) selectively die early in development, whereas females (ZW) die if cured of the *Wolbachia* infection following antibiotic treatment (Fig. 2). Studies of this system suggest that MK *Wolbachia* interferes with the sex-specific splicing pattern of the *Ostrinia* homologue of the sex determination gene *doublesex*, *Osdsx* (Sugimoto et al., 2010), producing a mismatch between the genotypic sex and expression of the phenotypic sex and leading to sex-specific death (Sugimoto & Ishikawa, 2012). Later examination of the levels of dosage compensation (Z-linked gene expression) in male and female embryos destined to die, revealed that misdirection of dosage compensation underlies the observed mortality. Males destined to die (from *Wolbachia*-infected females) have higher levels of expression of Z-linked genes than normal; while females destined to die (from females cured of the *Wolbachia* infection) have lower expression levels of Z-linked genes than normal (Sugimoto et al., 2015). In a related moth, *O. furnacalis*, RNA-Seq data of *Wolbachia*-infected embryos demonstrated that MK *Wolbachia* down-regulated a masculinizing gene, *Masc*, essential in controlling both sex determination and dosage compensation in Lepidoptera, compared to uninfected embryos. The decrease in *Masc* mRNA levels is reported to cause the MK phenotype via a failure of dosage compensation, and injection of in vitro transcribed *Masc* cRNA into *Wolbachia*-infected embryos rescued male progeny (Fukui et al., 2015).

The mechanism of MK in *Ostrinia* moths may be different to that underlying MK in other Lepidoptera. In *H. bolina* butterflies no female specific death is observed following antibiotic treatment to reduce or remove MK *Wolbachia* (Charlat et al., 2007a). It is interesting to note however, that the *doublesex* homologue in *H. bolina* may be involved in MK in this butterfly as it resides within the chromosomal region defined as containing a suppressor of MK action (Hornett et al., 2014). It would therefore be interesting to compare the *Wolbachia* strains and MK mechanisms of *Ostrinia* and *H. bolina*. Likewise, a comparison between the modes of action of the MK strain of *Wolbachia* in *Ostrinia* moths, which kills males as a consequence of feminising them through alteration of expression of *Osdsx*, and of ‘true’ feminising *Wolbachia* such as that infecting *Eurema* butterflies (see *Feminisation*) may shed light on how one genus of bacteria can induce multiple reproductive manipulations in their hosts and whether there is a functional link.

Finally, the Oriental tea tortrix moth *Homona magnanima* also carries a MK (Morimoto et al., 2001), however male death in this case occurs much later in development (termed ‘late MK’), and appears to be associated with two novel RNA sequences (Nakanishi et al., 2008). Late MK was originally only recorded in mosquitoes, with the causative agent being a microsporidian (Andreadis & Hall, 1979), however subsequent studies have now observed similar phenomena in other taxa including *Drosophila* flies (Jaenike, 2007). The extent of this type of manipulation, and the mechanisms underlying it, is still to be determined in insects, including Lepidoptera.

#### Feminisation

The feminisation of genetic males into functional phenotypic females (Stouthamer, Breeuwer & Hurst, 1999) is another strategy employed by maternally inherited endosymbionts to distort the host sex ratio towards the transmitting sex (females) (Fig. 1). While best known from the work on the association between *Wolbachia* and the terrestrial isopod *Armadillidium vulgare* (Juchault, Rigaud & Mocquard, 1992; Rigaud, Juchault & Mocquard, 1997; Bouchon, Rigaud & Juchault, 1998; Cordaux et al., 2004), feminisation also occurs in other female-heterogametic arthropods such as leafhoppers (XX/X0) (Negri et al., 2006), and Lepidoptera (ZZ/ZW). Other than *Wolbachia*, the Bacteroidetes bacterium *Cardinium* can also feminise males (Chigira & Miura, 2005; Groot & Breeuwer, 2006), however *Cardinium* has not yet been reported in butterflies or moths.

Observation of female-biased lines of pierid *Eurema* butterflies in Japan (Kato, 2000) led to the identification of a feminising *Wolbachia* in *Eurema mandarina* (formerly *E. hecabe* Y type) (Hiroki et al., 2002). *E. hecabe* (formerly *E. hecabe* B type) was later also discovered to carry a feminising *Wolbachia* indistinguishable from that of *E. mandarina*, thus suggesting that the infection transferred between the allopatric butterfly hosts via a shared predator or parasite, or via hybrid introgression between the species (Narita et al., 2011). When *E. mandarina* infected larvae were fed antibiotics to cure them of the infection, many of the adults emerged displaying sexually intermediate traits in their wings, reproductive organs and genitalia. Moreover, age at which antibiotics were administered was found to be important, with the highest level of intermediate sexual traits being exhibited when first instar larvae were treated. This work demonstrated that endosymbionts might continually influence and interact with their host (Narita et al., 2007) rather than have phenotypes that are effective only at a discrete time point in the lifecycle of the host.

The process of feminising in *E. mandarina* is more complex than originally thought. Against expectation, female butterflies infected with the feminising strain of *Wolbachia*, *w*Fem, had only one, paternally derived, Z chromosome. This was proposed to be due to meiotic drive against the maternal Z, preventing the formation of the expected ZZ feminised males. It was also suggested that *w*Fem lines have lost the W chromosome, and rely on *w*Fem for female development as curing the infection with antibiotics results in all-male offspring (Kern et al., 2015). Later work demonstrated that *Wolbachia* itself was responsible for the disruption of maternal Z chromosome inheritance in *w*Fem infected females, as well as the feminisation of female ZO individuals that have lost the female-determining W chromosome (Kageyama et al., 2017).

#### Cytoplasmic incompatibility

Perhaps the most commonly observed reproductive manipulation employed by endosymbionts in insects is CI. Unlike for MK or feminisation, the sex ratio of host populations infected by CI-inducing endosymbionts is generally not altered. Instead the symbiont induces an incompatibility upon mating between infected males and females of a different infection status (i.e. uninfected or infected with a different symbiont strain), leading to the death of all or a proportion of the offspring (Yen & Barr, 1971, 1973) (Fig. 1). This incompatibility is proposed to occur due to a modification of the infected male’s sperm that can be rescued when the female is similarly infected (mod-res mechanism). This specific rescue function is lacking in uninfected females or females carrying a different infection (Hoffman & Turelli, 1997; Charlat, Calmet & Mercot, 2001; Poinsot, Charlat & Merçot, 2003). Infected females therefore have a reproductive benefit of successfully producing a full complement of progeny (relative to uninfected females), when mated to uninfected or similarly infected males in the population. While *Wolbachia* is often described as the causative agent, *Cardinium* also has this ability (Hunter, Perlman & Kelly, 2003; Perlman, Kelly & Hunter, 2008).

Cytoplasmic incompatibility has been observed in a number of Lepidoptera including the Mediterranean flour moth *Ephestia kuehniella* and the almond moth *Cadra cautella* (Sasaki & Ishikawa, 1999). Interestingly, CI *Wolbachia* was discovered to lower the amount of fertile sperm transferred in *C. cautella* during second matings. However, no effect was shown on the amount of apyrene (non-fertile) sperm suggesting that *Wolbachia* may only target fertile sperm production (Lewis et al., 2011). Further work is required to expand our knowledge of CI mechanisms in Lepidoptera. Similarly to MK, CI *Wolbachia* are often observed at high frequency in Lepidoptera populations. In a study of seven Japanese populations of the pierid butterfly *Colias erate poliographus*, CI *Wolbachia* occurred at 85–100% prevalence. The high infection frequency was ascribed to strong CI (i.e. a high proportion of the progeny from an incompatible cross die) and perfect vertical transmission of the bacteria (Tagami & Miura, 2004; Narita, Shimajiri & Nomura, 2009). Where CI reaches very high frequency within a host population, the incompatibility between infected males and uninfected females is rarely observed as few females remain uninfected. However, selection for beneficial effects of infection and an eventual shift towards a mutualistic relationship between host and symbiont would remain.

Symbioses can be extraordinarily complex; individuals can carry multiple endosymbionts with differing phenotypes. For instance, *E. hecabe* butterflies carry a feminising *Wolbachia* strain, but also a second strain that causes CI (Hiroki et al., 2004). This was the first indication that different strains of *Wolbachia* could infect a single individual and cause different phenotypes. Host context is important in the expression of endosymbiont-induced phenotypes—one symbiont strain can have the ability to cause more than one phenotype, including reproductive manipulations that were originally assumed to be distinct from each other. This has been exemplified in the butterfly *H. bolina*: in populations where *H. bolina* has evolved suppression of the action of MK, surviving infected males are incompatible with uninfected females in the population, i.e. expression of the CI phenotype (Hornett et al., 2008). This finding indicates a potential functional or mechanistic link between the two phenotypes. However phenotypic switching between CI and MK through mutations cannot yet be ruled out. An intriguing possibility is whether feminisation is also mechanistically linked to CI and MK. Some evidence that may suggest this latter link is provided in studies of the moth *O. scapularis*. As mentioned above, male moths that die as a result of infection with MK *Wolbachia*, were found to carry the female isoform of a homologue of the sex-determining gene, *doublesex*, and hence were feminised prior to death (Sugimoto & Ishikawa, 2012).

Artificial transinfection of *Wolbachia* strains have provided further evidence of the relative importance of endosymbiont or host in determining the nature of the phenotype expressed. While in some cases transfer of *Wolbachia* from the natural host into a novel host did not alter the phenotype expressed (e.g. *Wolbachia* causes MK in the natural host *O. scapulalis* and in the transinfected host *E. kuehniella* (Fujii et al., 2001)), host context is important in others. Transfer of CI *Wolbachia wCau-A* from *C. cautella* to *E. kuehniella* resulted in the expression of MK in the novel host (Sasaki, Kubo & Ishikawa, 2002). The strength of the phenotype may also alter in the novel host: the level of CI induced by *Wolbachia* in the transinfected host *O. scapulalis*, was higher than that in its natural host *E. kuehniella,* indicating that host factors as well as endosymbiont strain are important in determining the phenotype expressed (Sakamoto et al., 2005).

### Impact upon host fitness

It is becoming increasingly evident that many heritable endosymbionts do not manipulate host reproduction, and yet are still maintained within the host population. Host-associated microbes are now thought to be commonly beneficial to their host. For an inherited endosymbiont, the trade-off between virulence and transmission can lead to a reduction in its pathogenicity towards the host, and evolution towards mutualism (Weeks et al., 2007). At the extreme end of the spectrum are the obligatory endosymbionts, which are necessary for host survival or reproduction. The growing number of cases include: *Wolbachia* required for oogenesis in the wasp *Asobara tabida* (Dedeine et al., 2001; Dedeine, Bouletreau & Vavre, 2005); *Wigglesworthia* bacteria acting as an obligate nutritional mutualist in tsetse flies (Aksoy, 1995); and *Buchnera* bacteria providing essential nutrients to aphids (Buchner, 1965).

Many more endosymbionts are facultatively (non-essentially) beneficial, with fitness benefits including increasing host survival (Fry & Rand, 2002) or fecundity (Vavre, Girin & Boulétreau, 1999; Weeks & Stouthamer, 2004). Studies of the beneficial effects of endosymbiont infection in the Lepidoptera provide an unusual example in *Parnassius apollo*. In one isolated population this near threatened butterfly regularly exhibits deformed or reduced wings; however, while 86% of normal winged butterflies are found infected with *Wolbachia*, this percentage drops to 30% in individuals displaying deformed wings, and 0% in individuals with reduced wings. Although this is suggestive of a protective role of *Wolbachia* in the ontogenetic development of the butterfly, further study needs to be carried out to prove causality (Łukasiewicz, Sanak & Węgrzyn, 2016).

Microbial endosymbionts can contribute to insect adaptation by providing complementary or novel metabolic capacities, allowing the insect host to exploit host plant nutritional resources. One such instance has been observed in the phytophagous leaf-mining moth *Phyllonorycter blancardella.* In this system, a bacterial endosymbiont, most likely *Wolbachia*, indirectly affects larval nutrition by manipulating the physiology of the host plant to create photosynthetically active green patches in otherwise senescent yellow leaves. The phenotype, termed ‘green-island,’ produces areas of leaf viable for host feeding in a nutritionally constrained stage of the lifecycle. Curing the larvae of endosymbionts resulted in the non-production of ‘green-islands,’ and consequent increased compensatory larval feeding and higher mortality (Kaiser et al., 2010). The mechanism behind green island formation involves increased levels of cytokinins (CKs), plant hormones important in plant senescence and nutrient translocation. *Wolbachia* have been shown to be involved in the release of CKs by the larvae, creating these nutritionally enhanced areas of leaf. Whether the CKs are bacterial-derived or produced by the insect in response to *Wolbachia* infection (or a combination of both) remains to be fully understood (Body et al., 2013; Giron & Glevarec, 2014). Several strains of *Wolbachia* from both A- and B-supergroups have been identified in 13 Gracillariidae leaf-mining moth species, while none were found in ancestral Gracillariidae. Acquisition of the green-island phenotype appears to have occurred several times independently across the Gracillariidae in association with different *Wolbachia* infections (Gutzwiller et al., 2015).

Generally, vertically inherited endosymbionts are unlikely to be maintained in host populations if they are highly costly. However, direct fitness or physiological costs of infection have been observed where the symbiont also manipulates host reproduction. CI *Wolbachia* are maintained in the host population despite reducing male fertility (Snook et al., 2000) or female fecundity (Hoffman, Turelli & Harshman, 1990) in *Drosophila* flies, or detrimentally affecting fecundity, adult survival and locomotor performance in the parasitoid wasp *Leptopilina heterotoma* (Fleury et al., 2000). Among Lepidoptera examples, presence of MK *Spiroplasma* in *D. chrysippus* in Kenya was negatively correlated with forewing length, suggesting that the bacteria may adversely affect development time or the growth rate of larvae (Herren et al., 2007). Presumably these physiological costs are counter-balanced by the reproductive manipulations employed by heritable endosymbionts, thus enabling the symbiont to persist. Although not covered in detail here, we note that in contrast, a symbiont that is also (or only) horizontally transmitted, can be highly detrimental to the host yet still be maintained in the host population. Indeed, host death may be its source of transmission to a novel host.

The gregarine protozoan infection, *Ophryocystis elektroscirrha*, of the Monarch butterfly (*D. plexippus*) is one of the most studied cases of direct fitness costs of symbionts in Lepidoptera. While it is not heritable in the sense of being intracellular, we include it here as it is passed vertically from mother to offspring via the surface of the egg. An infected female inadvertently coats her eggs with protozoan spores that cover the outside of her abdomen during oviposition. Newly hatched larvae ingest these spores while consuming the eggshell (McLaughlin & Myers, 1970). The parasite, which requires the adult host stage for transmission, rarely kills larvae or pupae under natural conditions, however the degree of virulence and transmission trade-off varies depending on the level of infection at the adult stage. Where individual *D. plexippus* butterflies carry high densities of the protozoa, they have both reduced survival and flight capacity compared to individuals with lower density infections (Altizer & Oberhauser, 1999; de Roode, Yates & Altizer, 2008; de Roode & Altizer, 2010).

### Symbiont-mediated protection

Although understudied in Lepidopteran systems, an exciting avenue of research in arthropods revolves around a symbiont’s ability to afford the host some level of resistance to its natural enemies, often through interference with pathogen or parasite replication or transmission (reviewed in insects in (Brownlie & Johnson, 2009)). This may be particularly the case for heritable endosymbionts, where symbiont and host fitness is inextricably linked—competing infections may elicit a response by the endosymbiont to protect the host, and thus simultaneously itself (Haine, 2008). Such symbiont-mediated protection has been documented in numerous taxa, particularly the Diptera, including recent studies demonstrating the ability of *Wolbachia* to supply their *Drosophila* host with anti-viral protection (Hedges et al., 2008; Teixeira, Ferreira & Ashburner, 2008; Martinez et al., 2014). Similarly, maternally transmitted *Spiroplasma* were found to protect *D. neotestacea* against the sterilising effects of a parasitic nematode (Jaenike et al., 2010), and enhance the survival of *D. hydei* parasitized by wasps (Xie, Vilchez & Mateos, 2010).

Symbiont-mediated protection appears to be extremely diverse. Aphids are host to a range of inherited symbionts, several of which provide protection against parasitoid wasp attacks (Oliver et al., 2003; Ferrari et al., 2004) or fungal infections (Ferrari et al., 2004; Scarborough, Ferrari & Godfray, 2005). In the European beewolf wasp, *Philanthus triangulum*, *Streptomyces* bacteria are stored in special antennae glands and deposited together with the egg in the oviposition chamber. The bacteria secrete antibiotics protecting the developing wasp larvae against fungal pathogens (Kaltenpoth et al., 2005). There is even some evidence that symbionts can protect their host from predators by producing toxic compounds. For example, a bacterial endosymbiont (that is both vertically and horizontally transmitted) closely related to *Pseudomonas aeruginosa* produces the polyketide toxin pederin, which protects *Paederua* beetle larvae from predatory wolf spiders (Kellner & Dettner, 1996; Kellner, 1999; Piel, Höfer & Hui, 2004). Furthermore, endosymbionts may have the ability to inhibit a range of pathogens by priming the host immune system (Braquart-Varnier et al., 2008; Moreira et al., 2009; Hughes et al., 2011), suggesting that symbionts can interact with, and alter integral components of, host biology.

Conversely, it is important to acknowledge that endosymbiont infection can also increase pathogen load. While *Wolbachia* confers protection against a variety of pathogens and parasites in a wide range of hosts, pathogen or parasite levels can also be enhanced by the presence of an endosymbiont (Hughes, Rivero & Rasgon, 2014): in the moth *Spodoptera exempta*, *Wolbachia* triggers a higher rate of virus infection and therefore lowers host fitness (Graham & Wilson, 2012).

### Host genetics

#### Host population genetics

Sex ratio distorting symbionts are likely to have a severe impact upon host population biology (Engelstädter & Hurst, 2007). If the prevalence of a sex-ratio distorter is high, the sex ratio of the population can become severely biased. In consequence, the hosts’ effective population size (*Ne*) will be reduced. Where there is little gene flow into the population (i.e. low immigration), a reduction of the effective population size may affect the amount of standing genetic variation and the potential for the host population to respond and adapt to environmental change. In contrast, if gene flow does occur, spatial variation in sex ratio (as seen in the butterfly *H. bolina* (Charlat et al., 2005; Hornett et al., 2009)) may result in asymmetric gene flow between populations. Although both sexes typically contribute equally to the gene pool of the next generation, immigration of an individual into a population in which the sex ratio is skewed against it (e.g. a male into a highly female-biased region) can have a much larger genetic impact (i.e. contribute more) than if that individual immigrated into an unbiased sex-ratio population (Telschow et al., 2006). MK symbionts are also thought to hinder the spread of beneficial alleles and facilitate the spread of deleterious alleles, due to constrained gene flow from infected to uninfected individuals within the population (Engelstädter & Hurst, 2007). In a further complication, strains expressing different reproductive manipulations may be incompatible. Although most famous for its MK *Wolbachia* infections, some populations of *H. bolina* also carry a CI-inducing strain of *Wolbachia*. This CI strain is phylogenetically distant from the MK strain, and crosses between MK-infected females and CI-infected males are fully incompatible with no progeny surviving. The incompatibility produced has led to strong competition between the two strains, with the CI-strain being able to not only spread successfully through uninfected populations, but to also resist invasion by the MK-strain carried by butterflies from neighbouring island populations (Charlat et al., 2006). Extending from this model, a study (Zug & Hammerstein, 2018) recently showed that when direct fitness benefits are taken into account in parallel to reproductive costs, the CI-strain is likely to also be able to spread across MK-infected *H. bolina* populations. Taken together, this suggests that successful establishment of particular butterfly genotypes is affected by the endosymbionts they harbour.

#### Linkage with host mitochondrial DNA

Maternally inherited symbionts residing within the cytoplasm of cells can alter the diversity and population genetics of the host’s mitochondrial genome (mtDNA). Co-inherited symbionts and mitochondria are in linkage disequilibrium, therefore when a cytoplasmic symbiont invades a population, the initially associated mitochondrial haplotype (mitotype) may ‘hitch-hike’ and correspondingly increase in frequency. Should such a selective sweep have occurred recently, the effective population size and genetic diversity of mtDNA would be reduced to that of the infected individuals (Johnstone & Hurst, 1996), and the geographic structure of mitochondrial variation lost. The latter has been observed in *Acraea* butterflies (Jiggins, 2003) and the comma butterfly *Polygonia c-album* (Kodandaramaiah et al., 2011). The tight association between endosymbiont and mtDNA can therefore seriously confound the results of any study using mtDNA genes as neutral genetic markers (Hurst & Jiggins, 2005). Reconstruction of phylogenetic trees using mitochondrial markers are hence likely to be misleading, particularly within shallower branches, when the study species is infected. In the Diamondback moth, *Plutella xylostella*, the main correlate of mtDNA variation is presence or absence of the *plutWB1 Wolbachia* infection (Delgado & Cook, 2009), and the lycaenid butterfly *Lampides boeticus* may have experienced accelerated population differentiation due to *Wolbachia* infection (Lohman et al., 2008). Recognition of these processes should lead to an increasing number of Lepidopteran studies interested in using mtDNA markers to systematically screen for maternally inherited symbionts.

Where there is perfect transmission of the maternally inherited symbiont from the host to its offspring, infected individuals all carry the same mitotype, while uninfected individuals remain polymorphic. This pattern has been repeatedly observed in natural populations of insects, including in the Lepidoptera. In the butterfly *H. bolina*, a strong association between one specific *Wolbachia* strain and one particular mitotype supported the hypothesis that the MK infection occurred with very high vertical transmission efficiency and rare horizontal transmission. In *H. bolina*, this strain of *Wolbachia* is thought to have undergone a recent selective sweep and was introduced into this butterfly through introgression, potentially from another *Hypolimnas* species, *H. alimena*. Conversely the infection and associated mitotype may have been introgressed from *H. bolina* to *H. alimena* (Charlat et al., 2009; Duplouy et al., 2010; Sahoo et al., 2018). Similarly, in the *Acraea* butterflies, a study of mitochondrial variants demonstrated that a MK *Wolbachia*, together with the associated mitotype, had introgressed from *A. encedana* into *A. encedon* within the last 16,000 years. As female butterflies are heterogametic (ZW), this event could potentially also lead to the introgression of genes on the female W chromosome (Jiggins, 2003). This scenario appears to have occurred in *D. chrysippus* infected with a MK *Spiroplasma*, as all infected females carry the same W chromosome variant (Smith, Gordon & Allen, 2010). This aside, the nuclear DNA is generally less likely to be in linkage with inherited symbionts. Gompert et al. (2008) studying North American *Lycaeides* butterflies reported that the spread of an endosymbiont (and associated mitotype) through the host population produced substantial mito-nuclear discordance. Therefore, the evolutionary history of an individual’s nuclear and mitochondrial genomes may be very different from each other. Such discordance may have far-reaching effects on host metabolism and physiology, as coevolution between nuclear and mitochondrial components of essential pathways is broken down.

#### Speciation by symbiosis

The concept that symbionts can be important promoters of speciation and diversity has been around for a long time (Wallin, 1927; Laven, 1959; Thompson, 1987; Breeuwer & Werren, 1990; Hurst & Schilthuizen, 1998; Bordenstein, 2003), but has recently been rejuvenated with the development of microbiome analyses (Brucker & Bordenstein, 2012). Contemporary evidence of microbe-assisted speciation involves pre-mating reproductive isolation through behavioural barriers such as mate preference, associated with the microbiome of the potential partners (Koukou et al., 2006; Miller, Ehrman & Schneider, 2010; Sharon et al., 2010; Chafee et al., 2011). The underlying mechanisms may involve alteration of the sex pheromones, interference with sensory organs, or effects upon immune-competence and hence mate attractiveness. Ecological isolation may also be heavily influenced by microbial symbionts. Although the genetic basis of niche or habitat specificity is widely accepted, there is also increasing evidence that symbionts may play a role in determining host resource availability (Akman et al., 2002; Hosokawa et al., 2010), and thus may facilitate niche separation.

Additionally, endosymbionts might enable host speciation through post-mating isolation. In particular, strong bi-directional CI may result in reproductive isolation between hosts carrying different CI symbiont strains (Hurst & Schilthuizen, 1998; Werren, 1998; Bordenstein, 2003). In order for speciation to follow CI, a stable infection polymorphism must be maintained across host populations. This has been demonstrated in many systems including the butterfly *H. bolina* (Charlat et al., 2006). Theory predicts that two bi-directional CI-inducing symbionts can be stable for even high migration rates (Telschow, Hammerstein & Werren, 2005). What is more contentious is that for speciation to occur, the CI produced must be very strong (i.e. no offspring surviving from such crosses), and the symbiont must be maintained at a high transmission rate over time, to allow significant nuclear divergence (Engelstädter & Hurst, 2009).

Male-killing has also been linked to speciation in the butterfly *D. chrysippus*. In Kenya two forms exist: *D. c. chrysippus* and *D. c. dorippus*, separated by a hybrid zone. Each subspecies has an individual colour pattern controlled by locus *C*, which is intermediate in the hybrid (*Cc*). The *C* locus lies on an autosome that has fused with the W chromosome within the hybrid zone, physically linking colour pattern with female determination. A locus on this same autosome has also been associated with susceptibility to MK by *Spiroplasma*. The hybrid zone is characterised by female-biased sex ratios, caused by MK *Spiroplasma* that infects *D. c. chrysippus* or hybrid females, but rarely *D. c. dorippus* females. As immigrant males into the hybrid zone are predominantly *D. c. dorippus*, gene flow between the two subspecies is restricted: *D. c. chrysippus*/hybrid female × *D. c. dorippus* male crosses produce female-biased broods (Smith et al., 2016).

#### Sex determination

The maternal inheritance of intracellular endosymbionts has led to a great degree of interaction of the symbiont with the sex determination pathways of the host (reviewed in (Cordaux, Bouchon & Grève, 2011; Kageyama, Narita & Watanabe, 2012; Ma, Vavre & Beukeboom, 2014) and so not discussed in detail here). Maternally inherited endosymbionts distort the host sex ratio in order to enhance the fitness of the transmitting female sex. The mechanisms underlying these phenotypes often require considerable manipulation of host sex determination. We have seen above that in several cases MK and feminising *Wolbachia* can interfere with central components of the sex determination pathways in Lepidoptera. When a feminising element is highly prevalent in a host population, sex determination may be inextricably linked to the presence or absence of feminising activity (Hiroki et al., 2002), but may also enter into conflict with other genetic elements not under similar maternal inheritance. Furthermore, evolution of host suppressors of feminisation may move the system away from the original ZZ/ZW sex determination system. In *E. mandarina*, *Wolbachia* disrupts the inheritance of maternal Z chromosomes in *Wolbachia-*infected females, and feminises the resulting Z0 individuals that have lost the female-determining W chromosome (Kageyama et al., 2017). The host may then be prompted to evolve a strategy to counteract the feminising effects of the symbiont. It has been speculated that in the pillbug *A. vulgare*, a masculinising factor in the form of a dominant autosomal *M* gene has evolved in the host to counter the effect of the feminising endosymbiont (Rigaud & Juchault, 1993; Caubet et al., 2000).

#### Evolution of host resistance

Co-evolution between a host and a detrimental symbiont can result in the evolution of host genetic modifiers of symbiont presence or action. Despite this, and considering the wide array of costly effects that endosymbionts can impose on their hosts, it is perhaps surprising that there are relatively few well documented examples of the host having evolved genetic resistance to an endosymbiont. Indeed no suppression of the detrimental phenotype is observed in several studies where it may have been expected (Hurst, Jiggins & Robinson, 2001; Veneti, Toda & Hurst, 2004; Dyer & Jaenike, 2005). However, artificial transinfection experiments have provided an indirect method of discovering whether a host has evolved resistance to an endosymbiont, and have suggested that suppression of reproductive manipulation phenotypes may actually be common. In the moth *C. cautella*, which is naturally infected with two *Wolbachia* strains (*w*CauA and *w*CauB), artificial transinfection of CI-inducing *w*CauA to a sister host species, *E. kuehniella*, resulted in the transferred bacteria inducing MK instead of CI in the novel host (Sasaki, Kubo & Ishikawa, 2002; Sasaki, Massaki & Kubo, 2005). By interpreting these data in the light of the hidden MK theory (where MK is masked by the presence of a fixed suppressor), this switch in phenotype between species could be interpreted as the ‘unmasking’ of MK when released into a background devoid of host suppression genes. More generally, resistance may also underlie the loss of infections from populations or host species, however this is obviously hard to document in nature.

The selective pressure for host resistance is particularly strong when the sex ratio is severely biased (Düsing, 1884; Fisher, 1930; Hamilton, 1967), and therefore one would expect the evolution of resistance particularly in cases of highly prevalent sex-ratio distorters. As mentioned above, the Samoan population of the butterfly *H. bolina* had an extraordinarily female-biased sex ratio of 100 females per male, caused by 99% of the females being infected with MK *Wolbachia* (Dyson & Hurst, 2004). However between 2001 and 2006 the dynamics of the interaction changed dramatically when *H. bolina* evolved suppression of the MK trait, allowing infected males to survive and rapidly re-establishing a 1:1 sex ratio within approximately 10 generations of the host butterfly (Charlat et al., 2007b). The presence of a zygotically acting dominant suppressor locus had previously been documented in SE Asian *H. bolina* populations (Hornett et al., 2006).

Sex ratio distorting endosymbionts can also have much wider implications upon host genetics. Recent work on the same Samoan population of *H. bolina* investigating the genomic impact of the rapid spread of suppression revealed that a substantial selective sweep had taken place, covering at least 25 cM of the chromosome carrying the suppressor locus. In addition to large changes in the frequency of genetic variants across this broad region, the sweep was associated with the appearance of several novel alleles. This suggests that the suppressor spread following migration of butterflies carrying the locus, potentially from SE Asia, rather than from a de novo mutation occurring within the population. It is also interesting to note that the suppressor of MK has been located to the chromosome containing *doublesex* (Hornett et al., 2014)—a sex determination gene demonstrated to be involved in *Wolbachia*-induced MK in *Ostrinia* moths.

#### Horizontal transfer of genetic material

While the extent of horizontal (lateral) gene transfer (HGT) between eukaryotes and prokaryotes remains uncertain, technological advances in genomics followed by an accumulation of microbial and host genomic data, have revealed that endosymbionts, particularly those that are vertically inherited, may readily exchange genetic material with their host. HGT from a prokaryote symbiont to its eukaryote host has been reported in many insects including beetles, flies, parasitoid wasps, mosquitoes and butterflies (Hotopp et al., 2007; Nikoh et al., 2008; Klasson et al., 2009; Werren et al., 2010) and has recently been reviewed in detail (Husnik & McCutcheon, 2018). Such movement of genes can afford the receiving organism important benefits. For instance, horizontally transferred bacterial DNA that is involved in the detoxification of cyanide has been identified in several moths and butterflies, allowing these insects to utilise otherwise noxious plants (Wybouw et al., 2014). However, the discovery of bacterial DNA within the host’s genome does not necessarily imply functionality, and definitive proof of function is difficult to obtain, indeed many transferred *Wolbachia* genes are not expressed at a significant level in the host (Hotopp et al., 2007; Nikoh et al., 2008). To date, the identification of a 350 bp long *Wolbachia* gene insert in the genome of the butterfly *Melitaea cinxia*, is the only reported example of an HGT from an endosymbiont to a Lepidoptera species (Ahmed, Breinholt & Kawahara, 2016), its origin and functionality have yet to be demonstrated.

Horizontal gene transfer is also known to occur in the opposite direction, from eukaryote host to symbiont. *Wolbachia* genome projects have indicated that genome fragments have been transferred from host to the bacteria, including in the *H. bolina* system. The MK *Wolbachia* strain sequenced appears to be extremely receptive to exogenous genetic material (Duplouy et al., 2013). In addition to cross-level transfer of genes, bacteria within a host may also exchange genetic material. Bacteria are known to be promiscuous with regard to DNA, with movement of bacteriophages between co-infecting symbiont species providing a convenient method of transfer of genes. Some endosymbiont traits are associated with phage presence (Oliver et al., 2009) and thus this movement offers the potential for transfer of traits between co-infecting symbiont strains (Duron & Hurst, 2013). Indeed, extensive HGT involving the bacteriophage *WO* has been reported between several *Wolbachia* strains infecting diverse hosts including within the Lepidoptera, Diptera and Hymenoptera (Masui et al., 2000; Bordenstein & Wernegreen, 2004).

### Behavioural modification

The transmission of many parasites is facilitated by their ability to manipulate the behaviour of their hosts (Lefevre et al., 2009). Reported cases are often restricted to viral and fungal pathogens; for instance, some baculoviruses and fungi cause summit disease—a syndrome that induce caterpillars to climb to high vegetation prior to being killed so that any spores released are carried further by the wind (Maitland, 1994; Yamazaki, Sugiura & Fukasawa, 2004). Behavioural modification of arthropod hosts by heritable endosymbionts is less evident, and where observed are perhaps more attributable to indirect effects of infection. *Rickettsia* bacteria have been associated with limiting long distance dispersal in a spider (Goodacre et al., 2009), and *Wolbachia* has been demonstrated to reduce wasp locomotor performance (Fleury et al., 2000). Models of MK endosymbionts in metapopulations have suggested that MKs can increase host dispersal rates (Bonte, Hovestadt & Poethke, 2008). These patterns may be attributed to the evolution of adaptive modifications by the symbiont to promote its own transmission. However another explanation is that these behavioural changes are merely side effects of physiological alterations without any adaptive causality.

In the butterfly, *D. plexippus*, the protozoan *O. elektroscirrha* has attracted much attention because of its potential involvement in the famous migratory behaviour of its host. This parasite is known to reduce the flight capacity of the host (Altizer & Oberhauser, 1999; Bradley & Altizer, 2005)—a trait that creates an important trade-off as the butterflies’ dispersive behaviour allows the spread of the protozoa across the species range, and thus increases the chance of it infecting naive populations. For the butterfly, migration offers an opportunity of escaping highly infected habitats where they may risk reduced fitness (Altizer, Bartel & Han, 2011). Altizer, Oberhauser & Brower (2000) demonstrated that variation in protozoa prevalence correlates with host movement—non-migratory populations have high infection prevalence whereas populations that migrate long distances show less than 10% prevalence of infection. More recently it was found that where migratory behaviour has been lost, the risk of infection is increased (Satterfield, Maerz & Altizer, 2015). Thus in part the presence of the protozoa may have led to Monarch butterflies forming both resident and migratory populations.

Further indirect behavioural consequences of microbial infection are also possible. In order to escape the fecundity and physiological costs of mating with an incompatible mate, individuals may evolve new adaptive mating strategies, including increased polyandry or mate discrimination (reviewed in (Miller & Schneider, 2012). *Wolbachia* influences mate-choice in the two-spotted spider mite, where uninfected females preferentially mate with uninfected males (Vala et al., 2004), while in *Drosophila paulistorum*, *Wolbachia* titer and mate discrimination are positively correlated (Miller, Ehrman & Schneider, 2010). In *Acraea* butterfly populations harbouring high frequency MK bacteria (thereby having highly female-biased sex ratios), infected females more often remained unmated than uninfected females (Jiggins, Hurst & Majerus, 2000). While this is suggestive of preferential mating by the male, further work needs to be carried out to test this. However, *Acraea* butterflies afford another example: in butterflies, males are often the competing sex and court the females. When the butterfly population is strongly female-biased due to the presence of a highly prevalent sex ratio distorting endosymbiont, the roles of the sexes may reverse. Such sex-role reversal was observed in *Acraea* butterflies infected with MK *Wolbachia*. Although male ‘hill-topping’ (swarming at the tops of hills) is common throughout the genus (Jiggins, 2002), in *A. encedon* the lack of males induced females to swarm instead, and to exhibit behaviours soliciting the males’ attention (Jiggins, Hurst & Majerus, 2000).

Male-killing endosymbionts may also result in female reproduction becoming sperm limited. In a comparison of *H. bolina* populations varying in MK *Wolbachia* prevalence, the prediction that female mating rates would decline with increasing MK infection prevalence as males became increasingly rare was not borne out. Unexpectedly the opposite occurs—as the population sex-ratio becomes more biased, the female mating rate increased until a point at which the lack of males makes it impossible for females to find a mate. It was suggested that female promiscuity increased in response to increasing male ‘fatigue.’ Males from more highly female-biased populations produced smaller spermatophores thus necessitating females to become more solicitous (Charlat et al., 2007c).

### Outstanding questions and future directions

We here are promoting the Lepidoptera as important models in the study of endosymbiont induced reproductive manipulations, with MK, feminisation and CI all being evident in butterflies and moths. Current research is uncovering the genetic and functional basis underlying these phenotypes but many outstanding questions remain: Are all three reproductive manipulations found in Lepidoptera functionally linked? How commonly can a single endosymbiont strain confer more than one phenotype? How do different endosymbiont genera confer similar phenotypes in their host (e.g. both *Wolbachia* and *Spiroplasma* cause MK in Lepidoptera), and are the mechanisms related? How does MK, feminisation and CI in Lepidoptera differ from that expressed in taxa with divergent sex determination systems? Also, how do sex-ratio distorting endosymbionts affect the long-term evolution of the host. Given recent advances in genomics this now can include investigations of the genomic impact of a sustained population sex-ratio bias. Sex-linked traits in particular may be expected to be affected.

More questions are provoked when research into heritable endosymbionts associated with other arthropod taxa is considered. Of particular interest is the evidence accruing that symbionts often afford the host some level of protection against pathogens and parasitoids. But how frequent is this phenomenon in butterflies and moths? Also, can we see these effects in combination with reproductive manipulations, producing a trade-off between detrimental and mutualistic effects of infection? Conversely, where we see highly prevalent and persistent endosymbiont infections in host populations that do not induce reproductive manipulations, do these symbionts offer the host protection? While there are clearly many outstanding questions to examine in the Lepidoptera, in this next section we focus upon four further areas of research that will move Lepidoptera-heritable endosymbiont research forward.

#### Comparative endosymbiont genomics

The genomes of many arthropod heritable endosymbionts have now been assembled, however very few of those sequenced are associated with Lepidoptera hosts. A comparative genomics approach can be used to elucidate endosymbiont evolution and function in its host including identifying candidate genes involved in reproductive manipulations such as CI (as in *Drosophila* (Sutton et al., 2014; LePage et al., 2017)), and parthenogenesis induction (in parasitoid wasps (Newton et al., 2016; Lindsey et al., 2016)). A recent comparison of 16 *Wolbachia* genomes identified a core *Wolbachia* genome of 496 sets of orthologous genes, 14 of which were unique to *Wolbachia* among the Rickettsiales bacteria, of which it is a member (Lindsey et al., 2016). This study included the MK *Wolbachia* strain *w*Bol1b from *H. bolina* butterflies, which was revealed to be closely related to a CI *Wolbachia* infecting *Culex pipiens* mosquitoes, *w*Pip. A comparison of the two strains identified a number of genes specific to *w*Bol1b that could be potential candidates involved in the induction of MK (Duplouy et al., 2013). An interesting future research direction that may inform on the diversity and genetic basis of MK, would be to expand this line of enquiry by comparing the genome of *w*Bol1b with other MK and non-MK *Wolbachia* genomes. Candidate loci could also be investigated in other MK-inducing symbiont genomes such as *Spiroplasma*. While the genomes of many *Spiroplasma* bacteria have been characterised from various arthropods (see (Bolaños, Servín-Garcidueñas & Martínez-Romero, 2015) for a minireview), including the MK *Spiroplasma* endosymbiont MSRO found in *D. melanogaster* (Paredes et al., 2015), to our knowledge none have as yet been published that specifically associate with Lepidoptera.

As high-throughput sequencing costs reduce, the genomes of increasing numbers of Lepidoptera are being sequenced (for a review of the current status see (Triant, Cinel & Kawahara, 2018)). A happy indirect consequence of this is that endosymbiont genome sequences can be retrieved as a by-product of host genome sequencing. This is a particularly useful tool when studying intracellular endosymbionts that are not readily culturable, and hence difficult to directly isolate and sequence (such as *Wolbachia*). This approach has been used to reconstruct the genome of *Wolbachia* infecting the moth *Operophtera brumata* (Derks et al., 2015), and that of *Wolbachia*, *w*Aus, associated with the moth *P. australiana* (Ward & Baxter, 2017). Interestingly, and similarly to *w*Bol1b from *H. bolina*, both strains were most closely related to the CI *Wolbachia w*Pip from the mosquito *C. pipiens* (Derks et al., 2015; Ward & Baxter, 2017); however, in the case of *w*Aus, two genes previously determined to be involved in CI caused by *Wolbachia* from *D. melanogaster* were not found in the genome of *w*Aus (Ward & Baxter, 2017). Further work needs to be conducted to characterise the nature of the interaction between *Wolbachia* and host before more insight can be gained through genomic comparisons.

#### What else is in there? Moving towards a metagenomics approach

This review has revealed a marked bias in Lepidopteran heritable endosymbiont research—*Wolbachia* is by far the most studied endosymbiont in butterflies and moths. While the incidence of *Wolbachia* is undoubtedly high in Lepidoptera and its effects upon its hosts important, the development of routine PCR assays and resources specific to this one genus of bacteria may have inflated its significance relative to other endosymbionts. Thus a practical limitation of the current methodology in the study of heritable endosymbionts in Lepidoptera is the lack of an unbiased approach to determine what microbes butterflies and moths carry. This is changing with the development of culture-independent methods of ascertaining what microbes, particularly bacteria, are present within an organism. High-throughput sequencing of the hypervariable bacterial 16S rRNA gene, and metagenomics allow the characterisation of whole bacterial communities of hosts. Particular to heritable endosymbiont research, attention should be given to the tissue from which DNA is sourced, as heritable bacteria are not necessarily found in the commonly sequenced gut tissue or lumen. Amplifying bacterial DNA from whole insects or the reproductive tracts may yield a clearer idea of the vertically inherited symbionts present. We also have to consider what constitutes a heritable endosymbiont; many Lepidopteran gut bacteria are transitory and/or environmentally acquired for example via the food plant as larvae (Mason & Raffa, 2014; Hammer et al., 2017), or nectar as adults and as such may not evolve symbiotically with the host. However, gut bacteria may be transmitted by the female to the progeny via for example the egg coating, which neonates often consume upon hatching. One challenge will be to distinguish which of the microbes present in a community are symbiotic, and further, which are vertically transferred. Therefore close behind microbiome characterisations of Lepidoptera will be experimental manipulations of the microbiome and the sequencing of progeny to ascertain heritability.

Revealing the microbiome of Lepidoptera will open up a new set of questions such as do gut microbes and heritable endosymbionts interact? Can endosymbionts affect the composition of the microbiome? Do their effects interact? One promising avenue of research is the antimicrobial activity of gut symbionts. The moth *S. littoralis* habours a gut bacterium *Enterococcus mundtii* that secretes an antimicrobial peptide (mundticin KS) against invading bacteria, but not against other resident gut bacteria. This antimicrobial activity directly inhibits competitors, but also potential pathogens, from the gut of its host. In *S. littoralis*, this extracellular symbiont persists across host developmental stages and is a major constituent of the microbiome across generations, suggesting that it can be vertically inherited, and that it may form a long-term symbiotic association with its host (Shao et al., 2017).

A further avenue for future research is the presence and impact of non-bacterial heritable endosymbionts. In particular there is increasing recognition that viruses may be vertically inherited and can have dynamic interactions with their host (reviewed in insects in (Longdon & Jiggins, 2012)). The moth *Helicoverpa armigera*, a crop pest, is infected with a vertically (and horizontally) inherited densovirus (HaDNV-1) that appears to be mutualistic. In wild larvae a negative interaction exists between the symbiotic densovirus and the presence of a nucleopolyhedrovirus (HaNPV) that is widely used as a pesticide against *H. armigera*. Laboratory work confirmed that larvae carrying HaDNV-1 had significantly higher resistance to the HaNPV pesticide, and also to low doses of *Bacillus thuringiensis* toxin. Additionally, HaDNV-1 infected individuals have a higher developmental rate and higher fecundity than that of their uninfected counterparts (Xu et al., 2014). In contrast, in the moth *H. magnanima* a novel RNA virus appears to be responsible for ‘late’ MK while being benign to female moths, thus acting as a reproductive manipulator (Nakanishi et al., 2008). Metagenomic sequencing has identified viruses across diverse arthropods (Li et al., 2015), and while often pathogenic a recent study identified a vertically inherited sigma virus in the nymphalid butterfly *Pararge aegeria*, that may have a more symbiotic role. In this species transmission of the virus was predominantly maternal (through eggs), with paternal (through sperm) transmission rates being much lower. Wild populations of *P. aegeria* experience high levels of infection, with a mean viral prevalence of 74%, and marked population structure in the genetic diversity of the virus (PAegRV). The nature of the relationship between *P. aegeria* and PAegRV remains to be determined (Longdon et al., 2017).

#### Global environmental change: can endosymbionts facilitate or constrain adaptation?

Predicting if or how organisms adapt to environmental change is a critical and timely question. Every organism interacts with a multitude of abiotic and biotic factors, including heritable endosymbionts, and knowledge of how these influence each other is imperative in understanding an organism’s adaptive potential. Global environmental change is likely to alter the level and direction of natural selection in host/symbiont co-evolution (Wolinska & King, 2009). In one direction, endosymbionts may increase the host’s potential repertoire for responding to environmental changes such as temperature, while we also recognise that the destabilisation of often finely tuned host-symbiont interactions may be severely detrimental for natural populations.

As poikilotherms—organisms that do not maintain internal thermal homeostasis—butterflies and moths are very susceptible to extreme temperatures (Denlinger & Yocum, 1998). While they utilise a range of mechanisms, including behavioural and physiological responses, to regulate temperature, every species is defined by thermal limits. Recent work has indicated that microbial symbionts of insects can often facilitate or constrain adaptation to environmental changes, including temperature. For instance, aphids carry symbionts that proffer heat stress protection (Montllor, Maxmen & Purcell, 2002; Russell & Moran, 2006), including a point mutation (a change in a single nucleotide), which governs host thermal tolerance (Dunbar et al., 2007). The temperature insects are exposed to during development is also important in the maintenance of symbionts (Anbutsu, Goto & Fukatsu, 2008), or to the phenotype expressed by the symbiont in the host (Hurst et al., 2000). With global environmental change, de-stabilisation of the host-symbiont interaction may become more frequent and have severe consequences for many species. The sudden loss of an obligatory mutualistic symbiont, for example, would almost certainly lead to a host population decline (for further discussion of host-symbiont interactions and temperature see (Wernegreen, 2012; Corbin et al., 2016; Moran, 2016).

Furthermore, the nature of the relationship between host and symbiont may be indirectly affected by the changing climate. A few degrees rise in temperature can alter the geographic range of Lepidoptera (Parmesan et al., 1999). For many species, such range shifts and colonisation events should only be possible if the plants they utilise were following a similar expansion, such as in the host-limited butterfly *Gonepteryx rhamni* (Gutiérrez & Thomas, 2000). Additionally, range shifts may lead to a switch in host plant species or increased generalisation (Braschler & Hill, 2007), bringing subsequent repercussions for Lepidopteran-endosymbiont interactions. For example, in the moth *P. blancardella*, where endosymbionts nutritionally benefit the host by creating photosynthetically active green patches in otherwise senescent leaves of the host plant (Kaiser et al., 2010), a shift in host plant use could make this ‘green-island’ strategy ineffective in a novel plant with a different chemical makeup. In contrast, novel host plant utilisation may also be facilitated by endosymbionts, including through enhanced provisioning of nutrients, or detoxification (reviewed in (Hansen & Moran, 2014)).

Finally, habitat degradation and fragmentation is likely to have several implications for natural host-symbiont dynamics. Habitat destruction has the effect of crowding insect populations into smaller patches, and through fragmentation and subsequent isolation, the amount of gene flow between populations becomes reduced. These factors may increase disease transmission within a population, and alter geographical variance in endosymbiont presence and prevalence.

#### Screening butterflies and moths of conservation concern for endosymbionts

The Lepidoptera are model organisms in the fields of conservation and climate change research. However, despite the high occurrence of endosymbionts in Lepidoptera, current conservation planning rarely includes data on endosymbiont infections of the species under consideration, a deficit that may profoundly influence the outcome of any management undertaken. For effective conservation, or to understand how species will respond and adapt to environmental and anthropogenic changes, it is important that we try to understand the intricate relationships that microbes have with the hosts in which they reside. Fortunately there is increasing recognition of this importance with several recent studies reporting endosymbiont infections in populations of endangered or near threatened Lepidoptera (Nice et al., 2009; Sakamoto et al., 2011; Patricelli et al., 2013; McHugh et al., 2013; Łukasiewicz, Sanak & Węgrzyn, 2016; Fenner et al., 2017). One study surveying 22 species of conservation concern (comprising members of the Lycaenidae, Nymphalidae, Hesperidae and Noctuidae) for *Wolbachia* found 19 to be infected (Hamm et al., 2014). Nice et al. (2009) examined the nature of a *Wolbachia* infection in the North American endangered Karner blue butterfly, *Lycaeides melissa samuelis*. Screening for endosymbionts revealed that across the western edge of this butterfly’s range there was a widespread *Wolbachia* infection. They went on to simulate demographic effects of the spread of *Wolbachia* into uninfected populations and suggested that the spread of such an infection might further reduce already small population sizes. The authors show concern that the *Wolbachia* infection was prevalent in many of the largest and least impacted populations of this butterfly. This is significant as these populations are likely candidates from whom captive propagation efforts would draw individuals, and so the chance of inadvertently infecting a naturally uninfected population is high.

Release of wild individuals or of those reared in captivity, either as part of conservation management schemes or for commercial purposes (birthdays or weddings), might have unexpected and undesirable impacts if not monitored correctly. Rearing Lepidoptera, which often occurs at high densities, can allow the accumulation of pathogens. Releasing these individuals back into the field may therefore alter the parasite load and consequent fitness of the receiving population. Movement of individuals between populations may also affect the natural spatial pattern of endosymbiont diversity and prevalence: novel microbes may be introduced, or symbionts that have locally adapted in the donor population may affect the host in dramatically different ways in the novel population or environment. Further consequences with regard to endosymbiont infection are likely to be numerous, for example competition between native and novel infections may result in a shift in the natural equilibrium between the host and its native microbes or the introduction of cytoplasmic endosymbionts may also introduce linked variants such as host mtDNA haplotypes or female-linked nuclear DNA. Furthermore, as we have seen in the butterfly *H. bolina*, movement of individuals could also introduce host resistance loci that irrevocably alter the dynamics of host-symbiont interaction, and may have a wider impact upon the host genome. In general, if a novel association does form and/or spread, there follows rapid evolution of both host and symbiont, with phenotypic alterations that alter or optimise the new symbiosis (for an example see (Weeks et al., 2007).

## Conclusion

The Lepidoptera have emerged as important models in the study of the genetic and functional basis of the reproductive manipulations heritable endosymbionts employ, particularly with regard to *Wolbachia* bacteria. The results of this cumulative work is suggestive of the role of endosymbionts in the evolution of host sex determination itself. We have no doubt Lepidopteran endosymbiont research will continue to highlight the omnipresence and importance of *Wolbachia* but we suggest that more attention should now be given to the presence and interaction of other heritable endosymbionts Lepidoptera carry. Metagenomic approaches enable an unbiased view of the microbial community residing within moths and butterflies, while comparative endosymbiont genomics may illuminate the genetic mechanisms underlying the phenotypes endosymbionts induce in their host. Finally, given the importance of Lepidoptera as key indicators of climate change and the growing numbers of species listed as endangered, the study of heritable microbial endosymbiont in the Lepidoptera should transition from being a pure science filled with interesting curiosities, to a necessity that will contribute to the preservation of natural biodiversity and inform conservation management.

## Supplemental Information

10.7717/peerj.4629/supp-1 Supplemental Information 1 Butterfly species infected with heritable microbial endosymbionts.

10.7717/peerj.4629/supp-2 Supplemental Information 2 Moth species infected with heritable microbial endosymbionts.

10.7717/peerj.4629/supp-3 Supplemental Information 3 References for Tables S1 and S2.

## Additional Information and Declarations

### Data Availability

The following information was supplied regarding data availability: The research in this article did not generate any data or code; this is a literature review.
